# Ocular motor and vestibular examination in the unconscious patient—standard of care

**DOI:** 10.3389/fneur.2026.1790158

**Published:** 2026-02-26

**Authors:** Alexander A. Tarnutzer, Aasef G. Shaikh, David S. Zee

**Affiliations:** 1 Neurology, Cantonal Hospital of Baden, Baden, Switzerland; 2 Faculty of Medicine, University of Zurich, Zurich, Switzerland; 3 Department of Neurology, Case Western Reserve University, Cleveland, OH, United States; 4 Neurological Institute, University Hospitals Cleveland Medical Center, Cleveland, OH, United States; 5 National VA Parkinson Consortium Center and Neurology Service, Louis Stokes Cleveland VA Medical Center, Cleveland, OH, United States; 6 Department of Neurology, School of Medicine, The Johns Hopkins University, Baltimore, MD, United States

**Keywords:** coma, dolls head maneuver, eye movements, minimally conscious state, ocular bobbing, ping-pong gaze, unresponsive wakefulness syndrome, vestibulo-ocular reflex

## Abstract

**Background:**

Eye movements play an essential role in the assessment of the unconscious patient and offer a window to the function of the brain. We review the range of ocular motor and vestibular findings in patients with impaired consciousness and present a practical approach to these patients.

**Methods:**

Based on a structured review of the literature (Pubmed, Embase) 54 suitable citations were identified amongst 4,241 total citations. A manual search of the reference list of selected papers added another 57 papers. Based on these publications the spectrum of eye movement abnormalities in the unconscious patient was characterized.

**Results:**

The pattern of eye movement abnormalities seen in the unconscious patient depends on the underlying cause and the extent/location of brain damage. Conjugate eye deviations may be observed with either supratentorial or infratentorial lesions, while disconjugate deviations may indicate superimposed eye muscle palsies or decompensated strabismus. The presence of a full range of spontaneous horizontal, oscillatory eye movements (e.g., ping-pong gaze) in the comatose patient usually indicates bilateral cerebral hemisphere dysfunction. With vertical spontaneous eye movements, the identification of a slower and faster phase helps to distinguish between nystagmus and ocular bobbing and its variants. Combined with absent reflexively-induced eye movements, typical ocular bobbing strongly suggests a structural pontine lesion, whereas other vertical spontaneous eye movement patterns do not predict specific (focal) damage. The reflexive eye movements, i.e., the vestibulo-ocular reflex (VOR), can be assessed in comatose patients either by head rotations, caloric irrigation or galvanic stimulation. Intact slow-phase responses indicate relatively preserved brainstem function and inability to keep the eyes in an eccentric position suggest a deficient velocity-to-position integrator either from brainstem or cerebellar involvement.

**Conclusion:**

Ocular motor and vestibular testing in unconscious patients offer a unique opportunity to assess both brainstem and cerebellar function and its interplay with higher cortical areas. It may also help predict outcome. Challenges to overcome include a lack of standardized diagnostic approaches to unconscious patients. Quantitative eye movement analysis, based on videooculography (VOG) and artificial intelligence using large multimodal data sets are promising new tools for diagnosis, longitudinal observational studies and prediction of outcome.

## 1 Introduction

Many conditions result in reduced vigilance and unconsciousness, including structural brain diseases such as intracranial hemorrhage or traumatic brain injury (TBI), and also diffuse neuronal dysfunction (e.g., due to non-convulsive status epilepticus, hypoxic encephalopathy after cardiac arrest or intoxications) and psychogenic unresponsiveness. While transient in some, loss of consciousness often requires management in an intensive care unit (ICU).

Disorders of consciousness are classically divided into coma, unresponsive wakefulness syndrome (UWS, previously referred to as vegetative state) and minimally conscious state (MCS), depending on the patient’s response to internal and external stimuli (1). When assessing coma, the level of consciousness, brainstem responses, motor responses and breathing patterns should be evaluated. Patients in coma have absence of arousal and awareness of one’s self and the environment lasting for more than 6 h by definition (2); thus they do not respond to internal (e.g., pain from injury, bladder fullness, thirst) or external stimuli (e.g., pain, touch, speech). Patients in UWS have a preserved arousal level, demonstrate sleep–wake cycles and 37–43% may show signs of awareness (3, 4). In MCS, cognitively mediated behavior occurs inconsistently, but is reproducible or sustained long enough to be differentiated from reflexive behavior (5). The level of consciousness is best expressed by the Glasgow Coma Scale (GCS) and the Full Outline of Unresponsiveness (FOUR) scale, which are the most standardized tools to assess the level of consciousnesses (6). The FOUR scale provides greater neurologic detail than the GCS and also incorporates brainstem function. It is especially useful in intubated patients (7).

For the diagnostic workup in those patients with prolonged and persistent unconsciousness, guidelines such as the one published 2020 by the European Academy of Neurology recommend a multimodal diagnostic approach including a standardized clinical evaluation, electroencephalography (EEG), and both structural and functional imaging (8). However, advanced diagnostic testing such as magnetic resonance imaging (MRI) may be limited by the patient’s poor general condition and by supportive devices such as ventilators. Thus, the bedside clinical examination in the unconscious patient is of great importance, allowing both a narrowing of the differential diagnosis, guided decisions on additional diagnostic testing and on prognosis (9). The neurologic exam is key in this diagnostic process [see, e.g., Christofi (7) for a review]. Here we will focus on the examination of the acutely unconscious patients though where relevant, we will discuss applications to the examination and evaluation of UVS and MCS. Amongst bedside examinations available, eye movement testing allows for an assessment of the integrity of both supratentorial (higher cortical) and infratentorial (brainstem, cerebellar) circuits. For example, conjugate eye movements rely on a functional network of neurons located in the brainstem (pons, midbrain) adjacent to the ascending arousal system that contributes to consciousness (10, 11). Ocular motor and vestibular testing in the unconscious patient therefore have a major role and can be considered as «a window to the function of the brain».

Here we review the range of eye movement abnormalities seen in the unconscious patient, with a focus on bedside and quantitative ocular motor and vestibular testing and the interpretation of findings and provide a practical approach how to work-up these patients. Furthermore, we will also discuss the prognostic value of eye movement abnormalities in the unconscious patient. Blinking and pupillary responses are not covered in this review, but may be found elsewhere (7, 9, 12, 13).

For this critical review a literature search (MEDLINE and Embase) was performed. The search strategy was designed by a clinical investigator with relevant domain expertise in neurology (AAT). We focused on English-language articles, using the following strategies with the following components: (1) defining the clinical state (i.e., coma, reduced vigilance), (2) examination techniques including bedside ocular motor / vestibular assessments or quantitative testing, and (3) defining the spectrum of eye movements studied. We also manually searched reference lists from eligible articles. We did not seek to identify research abstracts from meeting proceedings or unpublished studies. The literature search was last updated on September 9th 2025 and initially identified 4,241 unique citations. Based on a title and abstract review performed by AAT, a total of 110 papers were identified for full-text review and eventually 54 papers were considered suitable. Manually searching the reference list of selected papers resulted in another 57 papers that were reviewed. These papers provided the basis to determine currently reported practices, and to identify the range of ocular motor and vestibular findings seen in the unconscious patient. Thus, this paper reflects an extensive review of the current knowledge on eye movement testing in the unconscious patient and also identifies limitations and potential strategies to advance in the future.

## 2 The approach to the unconscious patient

In the examination of the unconscious patient the structured and detailed neurologic exam plays a key role as outlined in the seminal works of Fisher (12) and of Plum and Posner (9); thus a neurology consult is often ordered (14). The neurologic assessment of the unconscious patient covers a broad range, including the level of responsiveness by applying external stimuli, pupillary responses, spontaneous body movements, eye movements (both spontaneous and reflexive), corneal responses, muscle tone, autonomic functions, fundoscopy and otoscopy (7, 12, 15, 16) (see Table 1). Intubation, sedation and muscle relaxation can limit the neurologic examination, impairing the assessment of higher cortical functions including cognition, speech, language, motor function and the sensory system. Thus, the assessment of brainstem function [see, e.g., Benghanem et al. (17)] with a focus on eye movement testing usually offers the best opportunity to assess brainstem-cerebellar networks including the vestibulo-ocular reflex (VOR) and the gaze-holding circuits (9, 12, 18, 19).

**Table 1:** Bedside assessment of the comatose patient (7, 9, 12).

History taking (from relatives, friends, witnesses) ➔ ask about coma onset (abrupt vs. gradual) prodromal symptoms (headache, seizures, fever, stiff neck etc.…) medical history current medication use of (illicit) drugs/alcohol
General medical examination ➔ check vital signs, blood pressure, respiratory patterns, temperature, skin
Neurologic examination: Assess the level of unresponsiveness (GCS, FOUR) Check brainstem reflexes including pupillary responses, corneal responses, gag reflex, respiratory pattern Perform fundoscopy (papilledema indicating elevated intracranial pressure, hemorrhage) and otoscopy (otorrhea, hemotympanum indicating basal skull fracture) Perform eye movement testing (see below) Check muscle tone and deep tendon reflexes
Ocular motor testing (see also Table 3) Eye position ➔ look for conjugate / disconjugate gaze deviations in the horizontal / vertical plane Eye movements ➔ distinguish between spontaneous, reflexive, and voluntary eye movements Describe beating plane (horizontal/vertical/torsional or combined) Check for the presence / absence of a slow phase / fast phase Report eye misalignment, eye muscle palsies, limitation of gaze Vestibulo-ocular reflex (VOR) ➔ reflexive eye movement testing Bedside horizontal/vertical VOR Look for a compensatory slow phase ➔ confirms integrity of brainstem VOR Check for full range of ocular motility ➔ eye muscle or gaze palsies? Assess patient’s ability to keep gaze eccentric using the VOR to move eyes eccentrically ➔ integrity of velocity-to-position integrator?

GCS = Glasgow Coma Scale; FOUR = Full Outline of Unresponsiveness; VOR = vestibulo-ocular reflex.

### 2.1 The bedside eye movement examination

We will review a structured examination of ocular motor and vestibular domains that are accessible in the unconscious patient in the following sections, addressing eye movement abnormalities in the vertical and horizontal plane separately. Thus, this excludes ocular motor domains that depend on cortical visual processing and require the patient’s active participation such as voluntary saccades and pursuit. While visual tracking is absent in the comatose patient, visual fixation and visual following has been reported in those patients with UWS and MCS. Thus, we will also review these findings.

**Figure 1:**
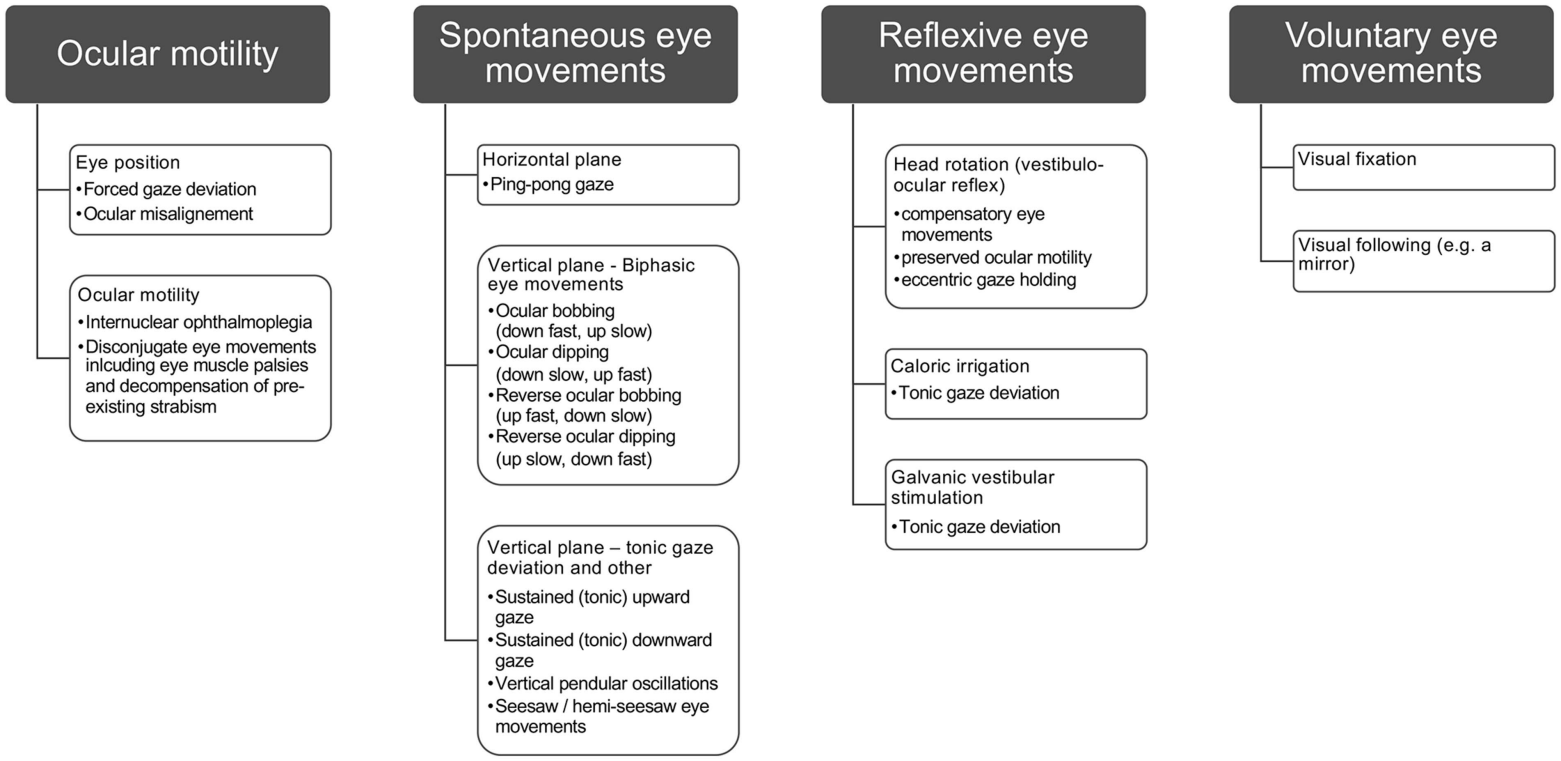
Range of eye movement abnormalities that can be seen in the unconscious patient, distinguishing different ocular motor domains (spontaneous/reflexive/voluntary eye movements) and assessing also ocular motility.

The eye movement examination in the unconscious patient should start by observing the position of the eyes in resting gaze (i.e., looking for a forced gaze deviation or ocular misalignment such as a skew deviation), looking for any ocular instability/spontaneous eye movements, and then testing the integrity of the horizontal and vertical VOR (see Table 1). Regarding the resting position of the eyes, one should note conjugate or disconjugate deviations from the straight-ahead position in the horizontal and/or vertical plane. When assessing eye movements, one should distinguish among spontaneous, reflexive and voluntary eye movements (see Figure 1). When observing spontaneous eye movements in the unconscious patient, epileptic seizures should be considered. In case of eye muscle palsies (e.g., third or fourth nerve palsy), monocular variants of spontaneous eye movements may be observed, as has been reported for ocular bobbing and ocular dipping (see below) (20, 21). External stimuli that are used to study the presence and range of reflexive eye movements in the unconscious patient focus on the VOR, elicited either by head rotations, caloric irrigation or galvanic vestibular stimulation (22). As a principle, fast corrective eye movements (quick phases or saccades) during testing of the VOR will be absent in the acutely comatose patient.

If disconjugate eye movements are observed in patients with impaired consciousness, consider potential underlying causes, including cranial nerve palsies, decompensation of pre-existing strabismus and coma-induced loss of control of vergence. For example, when applying the VOR at the bedside, the abducting eye may be lagging, indicating a sixth nerve palsy. A preserved full-range of vertical and horizontal eye movements during VOR, on the other hand, indicates an intact brainstem from the vestibular nuclei caudally to the ocular motor nuclei (III, IV, VI) rostrally (7, 12). If there is a complete ophthalmoplegia in an unconscious patient, one must also consider an acute demyelinating polyneuropathy and a neuromuscular block due to drugs or botulism.

Note that conjugate gaze deviations in the unconscious patient may be related to various conditions, including frontal lobe damage and brainstem lesions. For frontal lobe damage, eye deviation is towards the affected side in case of destructive lesions or towards the unaffected side in case of hyperexcitability (e.g., due to seizures) and with reflexive eye movements being preserved. Thus, a vestibular stimulus can usually drive the eyes across the midline notably, for seizures, eye deviations are typically intermittent and accompanied by a head turn also. Conjugate horizontal deviations away from the side of the lesion are typically observed in pontine lesions, but sometimes also in thalamic lesions (“wrong way deviations”).

Drug intoxication may abolish spontaneous or reflexive eye movements in the unconscious patient. For example, in a single patient in “light coma” due to amitriptyline intoxication, reflexive eye movements during caloric irrigation and VOR-testing were absent (23). Likewise, external ophthalmoplegia has been reported in patients receiving phenytoin and primidone for treating seizures and varying level of reduced consciousness (24, 25), with full recovery of ocular motility after cessation of phenytoin.

#### 2.1.1 Assessing the vestibulo-ocular reflex in the unconscious patient

The anatomical structures generating vestibular nystagmus are mainly located in the brainstem and are affected by consciousness. Thus, the pattern of vestibular responses helps distinguish among coma caused by metabolic alterations, structural lesions in the brainstem, and structural lesions in the cerebral hemispheres (18). Previously used terms such as “doll’s head maneuver” or “oculocephalic reflex” refer to assessing vestibular responses by passive head-on-body rotations in the horizontal or the vertical plane. However, these terms may be misleading and should be discarded in favor of the “vestibulo-ocular reflex” for describing testing of the three-neuron reflex arc when passively stimulated by head rotation and assessing compensatory eye movements to stabilize eye position (gaze) in space (18).

For assessing the vestibulo-ocular reflex (VOR) the patient’s head is rotated either along a horizontal or vertical axis. This should be applied cautiously in the ventilated patient, making sure that that tube is not disconnected. Furthermore, clearance from trauma surgery for head manipulation must be obtained in patients with traumatic brain or neck injuries or falls. What can be considered a “normal” VOR response depends on the patient’s state of vigilance. When the head is rotated in a conscious patient the VOR ensures steady fixation on an object of interest by generating compensatory eye movements in the orbit with a negligible delay of about 8–12 ms. The response seen in the unconscious patient is much different. This can be explained by the anatomy of the VOR as discussed below.

When applying the VOR and interpreting eye movement responses, it is important to consider the physiological response and which prerequisites are necessary for a normal response (see Table 2) (18). When testing the VOR, the patient’s head may be moved either rapidly from one position to the other or the head may be oscillated back and forth. A compensatory slow phase of nystagmus indicates the presence of a vestibular response and thus a structurally and functionally intact pontine or mesencephalic reticular formation (previously referred to as “positive doll’s head maneuver”). A resetting quick phase, however, in addition also requires alertness, thus its presence also excludes a significant depression of the level of consciousness in the acutely affected patient and thus integrity of the reticular activation system (18). Lack of quick phases is a non-localizing sign, indicating either direct damage to the reticular activation system or to descending supratentorial pathways. In contrast, unilaterally lacking quick phases imply a focal (usually posterior-fossa) structural lesion. Importantly, quick phases may return with the emergence of a UWS.

**Table 2:** Angular vestibulo-ocular reflex (VOR) testing – interpretation of findings (18).

Question asked	Indicative of
Is there a VOR slow phase and is it fully compensatory for the head turn?	Presence of a normal vestibular response
Is there a resetting quick phase?	Structurally intact pontine or mesencephalic reticular formation (and thus excludes a significant depression of the level of consciousness). If unilaterally absent ➔ focal infratentorial lesion.
If there are no quick phases, can both eyes be driven into extreme contraversive positions in the orbit?	Intact abducens and oculomotor nuclei as well as a functionally preserved medial longitudinal fascicle (INO).
Can the eyes be held in an eccentric position in the orbit if rotation of the head is stopped when the eyes are fully deviated in the orbit?	Intact brainstem/cerebellar gaze-holding network (and its absence indicates depressed brainstem function)

INO = internuclear ophthalmoplegia; VOR = vestibulo-ocular reflex.

In the case of absent quick phases, the range of compensatory head movements during VOR testing should be assessed. Specifically, it should be tested if eye movements are fully compensatory, i.e., if the eyes can be driven into the extreme contraversive positions in the orbit. This indicates intact abducens and oculomotor nuclei as well as a preserved medial longitudinal fascicle that is forwarding the commands to the ocular motor nuclei. In a next step, the patient’s ability to keep the eyes in an eccentric position should be assessed. Therefore, the rotation of the head is stopped in a position where the eyes are fully deviated in the orbit. Eccentric gaze holding is mediated by a brainstem-cerebellar network referred to as the oculomotor velocity to position integrator. Malfunction of this neural network, i.e., insufficient eccentric gaze holding during the VOR in the comatose patient, is a non-specific sign of impaired brainstem/cerebellar function (18). While in healthy subjects rotations of the trunk relative to the stationary head elicits a negligible response cervical ocular responses may be potentiated in patients with pre-existing, chronic bilateral vestibular loss-of-function (26). This emphasizes the importance of knowing pre-existing medical conditions.

#### 2.1.2 Eye movement abnormalities in the horizontal plane—ping-pong gaze and related findings

We now assess ocular stability in the horizontal plane. Conjugate or disconjugate horizontal, short-cycling, periodic alternating gaze (without associated head movements) lasting hours to days in unconscious patients was first described by Fisher (27) (Table 3). Various terms have been used to characterize these periodic eye movements without pausing in the lateral positions, including “pendulum-like eye-oscillations” (28), “to and fro excursions,” “roving eye movements” (27) and “ping-pong gaze” (PPG) (29). The duration of cycles is reported to be 1.5 to 8 s (30, 31) demonstrating a triangular or pendular waveform (32). Typically, these alternating eye movements appear smooth, but saccadic patterns have also been reported (33, 34). PPG may be restricted to one hemifield, being linked to unilateral or strongly asymmetric bilateral hemispheric damage (31, 35).

**Table 3:** key features of eye movements in the unconscious patient (49, 113).

Type of eye movement	Beating characteristics	Speed	Brain areas affected	Prognosis	Comments
Horizontal plane					
Short-cycling periodic alternating gaze without pausing at lateral positions (“Ping-pong gaze”)	Horizontal, conjugate/disconjugate, to-and-fro movements, usually smooth, but sometimes saccadic.	Low-frequency (1.5–7 s per cycle)	Extensive supratentorial lesions (ischemic, metabolic, hemorrhagic, traumatic), cerebellar vermis lesions, metabolic-toxic (hepatic encephalopathy, hypoglycemic coma, myxedematous coma, MAO intoxication)	Usually unfavorable, favorable in selected patients without structural lesions	May be restricted to one hemifield in asymmetric hemispheric lesions or lateralized lesions.
Vertical plane (49, 53)					
Ocular bobbing	Fast conjugate downward eye movement followed by slow upward return to mid-gaze position	Low-frequency	High localizing value: mainly focal pontine lesions if horizontal eye movements are abolished (“typical ocular bobbing), but also pontine compression due to cerebellar hemorrhage possible. Unreliable for localization if horizontal eye movements are preserved (“atypical ocular bobbing”)	Outcome usually poor for typical ocular bobbing, more favorable for atypical bobbing.	(Reflexive) horizontal eye movements typically abolished
Reverse ocular bobbing	Fast conjugate upward eye movement followed by slow downward return to mid-gaze position	Low-frequency	Unreliable for localization. May occur with metabolic disorders (e.g., hypothyroidism)	Variable prognosis. May show good recovery in cases with metabolic disorders	(Reflexive) horizontal eye movements typically preserved.
Ocular dipping (“inverse bobbing”)	Slow conjugate downward eye movements followed by quick return to mid-gaze position	Low-frequency (6–10 s per cycle)	Unreliable for localization. Follows hypoxic–ischemic insult or metabolic disorder (renal/hepatic encephalopathy, Wernicke’s encephalopathy, CJD, post status epilepticus)	May show good recovery in renal/hepatic failure (49)	(Reflexive) horizontal eye movements typically preserved. May be combined with horizontal roving eye movements (PPG)
Reverse ocular dipping (“converse bobbing”) (49)	Slow conjugate upward eye movements followed by quick return to mid-gaze position	Low-frequency	Unreliable for localization		(Reflexive) horizontal eye movements typically preserved. May be observed in alert patients as well.
Vertical “myoclonus”	Vertical pendular oscillations (2–3 Hz)		Acute pontine lesions		
Small amplitude mainly vertical eye movements	Small, intermittent, rapid, vertical, horizontal or refractory movements		Pontine or midbrain destructive lesions, with coexistent seizures		
Seesaw/hemi-seesaw eye movements	Alternating half-cycles of elevation and intorsion of one eye concurrently with the depression and extortion of the fellow eye and vice versa		Pontomedullary or mesencephalic lesions	Usually poor prognosis due to structural damage	May be combined with atypical ocular bobbing
Sustained upward gaze	Persistent upward deviation of the eyes	Non-phasic, tonic	Diffuse cerebral and cerebellar damage with sparing of upper brainstem due to hypoxic encephalopathy	Usually poor prognosis due to structural damage	Vertical VOR usually unable to overcome the sustained upgaze
Sustained downward gaze	Persistent downward deviation of the eyes	Non-phasic, tonic	Hypoxic encephalopathy, diffuse subarachnoid hemorrhage		

CJD = Creutzfeldt Jakob disease; MAO = monamine oxidase inhibitor; PPG = ping-pong gaze.

Extensive bi-hemispheric structural lesions due to ischemic strokes, cerebral hemorrhage, hypoxic encephalopathy due to cardiac arrest, intravascular lymphoma or head trauma have been linked to PPG. However, lesions of the cerebellar vermis (albeit likely also with widespread anoxic brain damage (29)) and acute metabolic disorders have been observed in patients with PPG. This includes hypoglycemic encephalopathy/coma with widespread subcortical diffusion restrictions (28, 36), myxedematous coma (37), hepatic coma (27, 38) and drug intoxications with monoamine oxidate inhibitors (39–41) and in combination with neuroleptics (42). Usually, response to the VOR is lacking in these patients (41), but may be preserved in selected cases (37, 43). Interestingly, external stimuli have been noted to modulate PPG. For example, painful stimulation of the limbs or the face has been reported to result in the transient cessation of PPG in single cases (32).

PPG, while usually observed in patients with coma, rarely may also be seen in patients with preserved consciousness (31, 32). The mechanism of PPG is not understood and a number of unproven hypotheses have been proposed. For example, disconnection of the cerebrum from the horizontal gaze centers in the brainstem has been suggested (30, 32), leading to preserved brainstem horizontal gaze mechanisms with lacking cortical inhibition (32). A disinhibited velocity storage mechanism (34) and inherent rhythms in the oculovestibular system reserved for sleep states (44) may then lead to rhythmic oscillatory eye movements in the horizontal plane. Others have proposed that PPG reflects the paralysis of all saccade generating neurons, but leaving brainstem nuclei involved in the generation of pursuit eye movements intact (45). PPG may also reflect the release of search mechanisms that scan the environment for new visual information in intact individuals.

In general, patients who show PPG have unfavorable outcomes (36, 46), but good recovery with reversible conditions has been described in a single case series (31) and in a recent systematic review of the literature (28). These patients included those recovering from diffuse cerebral ischemia caused by hypotension after heart surgery (but without acute structural cerebral lesions) (30) or with hypoglycemic encephalopathy (28) or hepatic encephalopathy (38). In other circumstances, such as with infectious disorders (as, e.g., Eppstein-Barr virus encephalitis), postictal state (31) and after a suicidal attempt (28) PPG subsided when those patients regained consciousness. Likewise, after recovery PPG was reversible in patients with myxedematous coma (37), drug intoxication with monoamine oxidase inhibitors (39–41) or in combination with neuroleptics (42).

Importantly, PPG must be differentiated from periodic alternating gaze deviations, where lateral gaze is held for several minutes before slowly moving to the opposite lateral gaze and repeating the cycle (30, 47). This phenomenon is a variant of periodic alternating nystagmus due to lesions of the cerebellar nodulus, but without the resetting quick phases. Furthermore, in some patients with UWS spontaneous small amplitude repetitive saccades can be observed, resembling square-wave jerks (48).

#### 2.1.3 Eye movement abnormalities in the vertical plane—ocular bobbing/dipping and related patterns

In a next step we observe ocular stability in the vertical plane. Various eye movement abnormalities have been reported in the comatose patient in the vertical plane including both biphasic (i.e., being rapid in one direction, and slow in the opposite direction) patterns of spontaneous eye movements (49, 50) and tonic gaze deviations (51) (Table 3). They may present both together or each individually. When assessing the comatose patient for spontaneous vertical eye movements, the direction of the fast phase and the slow phase and the sequence of eye excursions is important. Applying horizontal vestibular stimuli such as the VOR (50) or caloric irrigation (20, 52, 53) may elicit abnormal, spontaneous vertical eye movements in coma. Forced downward gaze after caloric irrigation has been observed in comatose patients with drug overdoses, suggesting a search for a sedative-hypnotic drug (such as barbiturates) overdose as the cause of an otherwise unexplained coma (54).

##### 2.1.3.1 Ocular bobbing

**Figure 2:**
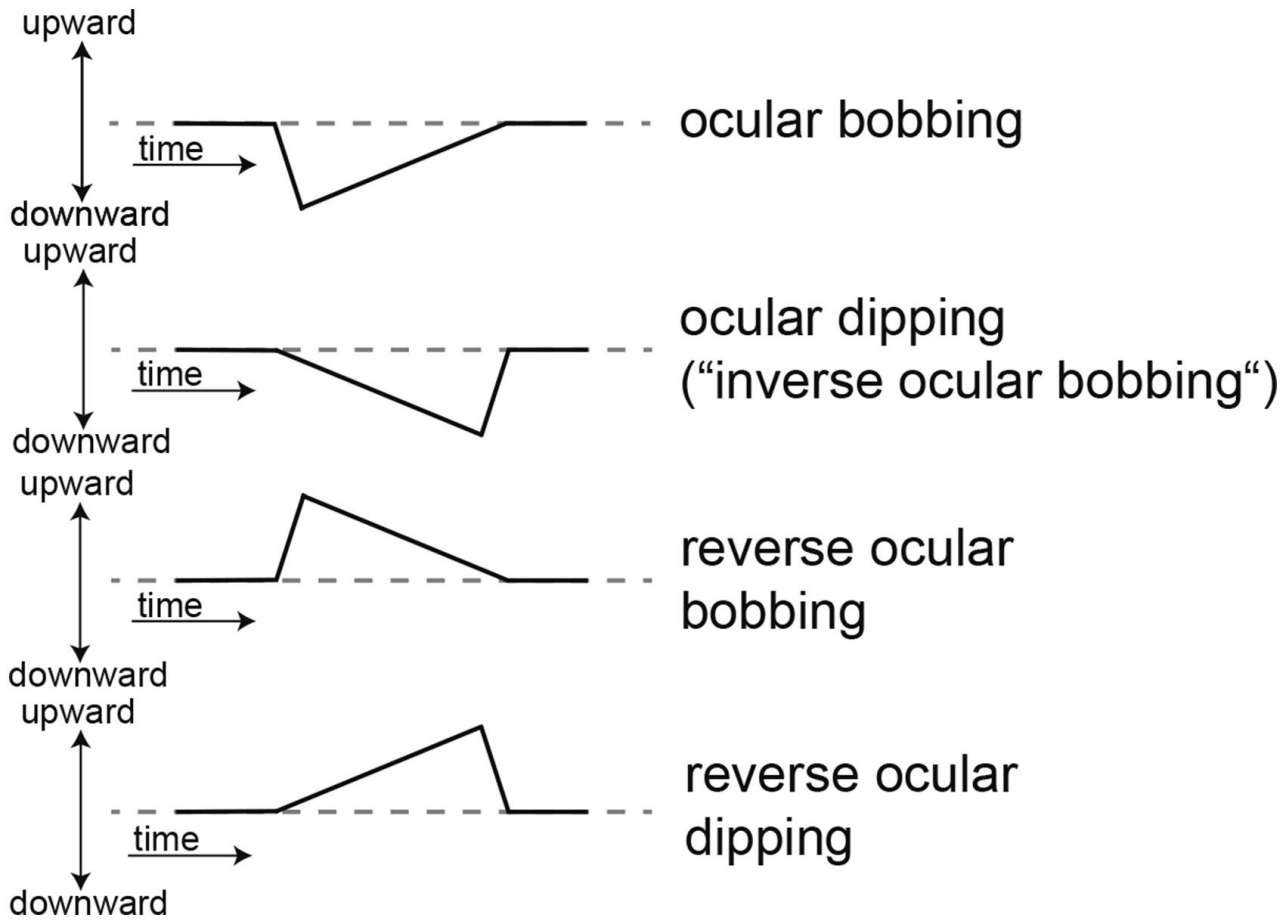
Schematic illustration of biphasic spontaneous eye movement abnormalities in the vertical plane, ranging from ocular bobbing and dipping to reverse forms. For all illustrations vertical eye position is plotted against time. A dashed line indicates horizontal eye position.

Fast downward conjugate eye movements with a slow return to primary gaze in unconscious patients have been referred to as “ocular bobbing” (55, 56) (see Figure 2). Pontine lesions (vascular, neoplastic) have been linked to ocular bobbing (52, 53, 55), and with absent reflexive horizontal eye movements (referred to as “typical ocular bobbing”) are often considered pathognomonic for an intrinsic pontine lesion (20, 57, 58). But exceptions have been described with typical ocular bobbing resulting from cerebellar hemorrhage and subsequent pontine compression (20, 59) or due to viral encephalitis (60). In patients with ocular bobbing in whom horizontal spontaneous and/or reflexive eye movements are (partially) preserved (referred to as “atypical ocular bobbing”), there is no localizing value and non-structural causes are more likely. This includes metabolic-toxic encephalopathies and obstructive hydrocephalus (49, 56, 61), intoxications with, e.g., organophosphates (62, 63) or bromide (64). However, more localized pontine ischemic (i.e., non-hemorrhagic) lesions (57) and pontine myelinolysis (65) have been reported with atypical ocular bobbing. Atypical ocular bobbing may also be associated with a locked-in syndrome (53). A monocular form of ocular bobbing may be observed in cases with contralateral third nerve palsy (56). Rarely, ocular bobbing has been reported in conscious patients with pontine hemorrhage or infarction (56–58, 66).

A combination of ocular bobbing and vertical pendular oscillations has been described in a single case and several potential mechanisms were discussed, including oscillations originating from a hypersynchronized, disconnected inferior olivary nucleus, an unstable vertical neural integrator of gaze-holding or low-frequency saccadic intrusions (67). While eye movements are usually conjugate in ocular bobbing, single cases with a disconjugate, seesaw-pattern have been reported. An intermittent non-pendular seesaw pattern was described in combination with atypical ocular bobbing in a patient with severe pontomedullary tegmental damage after a car accident (68). Another patient presented with a seesaw-pattern ocular bobbing and absent horizontal reflexive eye movements due to extensive pontine hemorrhage (69).

Damage of omnipause neurons in the pons, that function to inhibit extraneous, unwanted saccades enabling steady fixation, or disruption of cortical (supranuclear) control of omnipause neurons in cases with diffuse cortical dysfunction have been proposed as a potential mechanism of ocular bobbing (21). As such, ocular bobbing could be considered a type of saccadic intrusions (49). Functional brainstem impairment was proposed in a single case presenting with ocular bobbing and hepatic encephalopathy based on bilaterally abnormal brainstem auditory evoked potentials (61). In two patients with hypoxic encephalopathy, the EEG demonstrated generalized periodic discharges time-locked with ocular bobbing or dipping (70). This led the authors to conclude that ocular bobbing/dipping occurring in the setting of hypoxic encephalopathy may represent a myoclonic counterpart and thus should trigger an EEG evaluation as it may be the single sign of post-anoxic myoclonus.

Albeit the overall prognosis for ocular bobbing in comatose patients is poor, recovery after typical (60, 66) and atypical (62, 64, 71) ocular bobbing has been reported in single cases. Since atypical ocular bobbing is more likely with non-structural lesions; its prognosis is better than for typical ocular bobbing which is usually associated with a structural lesion.

##### 2.1.3.2 Ocular dipping (“inverse ocular bobbing”)

Ocular dipping (also called “inverse ocular bobbing”) is defined as a slow conjugate downward eye movement followed by a quick return to primary gaze (72, 73) (see Figure 2). It is rare and linked to hypoxic encephalopathy (73–76) and post status epilepticus (50, 72). Ocular dipping may also be seen in patients with diffuse cortical dysfunction related to Creutzfeldt Jakob disease (77), multifocal supratentorial hemorrhage (59, 78), Wernicke’s encephalopathy, encephalopathy related to hemiplegic migraine with confirmed CACNA1A mutation (79), anti-NMDA receptor encephalitis (80) and renal and/or hepatic failure (49). Thus, brainstem function is typically preserved and reflexive horizontal eye movements can be elicited. An increase in frequency and velocity of ocular dipping was observed in several patients after painful stimulation (21, 72), probably depending on the arousal pathways connecting the cortex, basal ganglia and brainstem (21). Horizontal roving eye movements, consistent with PPG (78) or similar to PPG (72, 75) were reported in several cases.

For further diagnostic work-up and for prognosis the distinction between ocular bobbing and ocular dipping is important. The first pattern makes focal pontine damage and thus poor outcome more likely (especially when accompanied by horizontal gaze palsy), whereas the latter pattern has been associated with better outcome due to diffuse cerebral dysfunction rather than focal lesions (49). Recovery after ocular dipping has been reported (21, 49, 53), with the spontaneous vertical eye movements stopping when regaining consciousness.

##### 2.1.3.3 Reverse ocular bobbing and reverse ocular dipping

For both ocular bobbing and ocular dipping reverse forms have been reported, which by definition are initiated by an upwards movement of the eyes (Table 3 and Figure 2). For reverse ocular bobbing a fast upward conjugate eye movement is followed by a slow downward return to mid-gaze position, whereas for reverse ocular dipping a slow upward conjugate eye movement is followed by a fast downward return to primary gaze (49). Reverse ocular bobbing has been linked to diffuse cerebral damage and has reported in cases with myxedematous coma (37) with reflexive horizontal eye movements preserved. Reverse ocular dipping has been reported in a case with focal pontine ischemic lesion and preserved consciousness (59). In individual patients more than one type of vertical spontaneous eye movements in coma may be seen (50).

##### 2.1.3.4 Tonic vertical gaze deviations in the comatose patient

Tonic vertical gaze deviations have also been described in comatose patients, being retrospectively identified in 56% in a consecutive case series in a single tertiary hospital (51). Diffuse cerebro-cerebellar damage sparing the brainstem has been postulated by the authors. Johkura and colleagues proposed that upward gaze deviation is considered an early sign, whereas downward gaze deviation appears later and generally implies a transition to a UWS (51).

Prolonged tonic upward gaze has been reported in with hypoxic encephalopathy; (51, 81, 82) presumably the upper brainstem was preserved (12). This tonic upgaze deviation could not be overcome by vertical VOR in one series of six cases (81), whereas VOR responses was preserved in another case (82). Disinhibition of the upward vertical VOR-pathway due to floccular disinhibition and lack of correcting saccades due to cerebral dysfunction has been proposed as the underlying mechanism of tonic upgaze deviation in coma (82). Over the course of disease, downbeat nystagmus may develop as the tonic upgaze deviation resolved (81), suggesting recovery of cerebral cortical function and its influence on generating saccades by the brainstem (82). It may also rarely be accompanied by tonic uninhibited elevation of the lids (referred to as “eyes-open coma”), related to pontomesencephalic dysfunction (83).

Sustained (tonic) downgaze has been reported in comatose patients with medial thalamic hemorrhage, diffuse subarachnoid hemorrhage and hypoxic and metabolic encephalopathy (84, 85), which may evolve into ocular dipping (85) and may be accompanied by PPG (84). Occurring later (usually 1–7 days after the initial event), tonic downgaze was considered a sign of partial recovery with changing balance in cerebellar projections to the brainstem or of brainstem pathways themselves (84). Prognosis is worse for patients presenting with sustained downgaze compared to those with upgaze. Twenty percent of patients with tonic upgaze regained consciousness in a large case series, whereas sustained downward gaze was uniformly associated with a poor prognosis and subsequent UWS (51).

##### 2.1.3.5 Other vertical eye movements in the comatose patients

Rarely, large-amplitude, vertical pendular oscillations (sometimes called “myoclonus”) may be seen in pontine stroke, usually accompanied by horizontal gaze palsy (86). Small-amplitude, vertical eye movements may be the only finding in patients with epileptic seizures and co-existent brainstem damage (87). Furthermore, subtle vertical eye movements may also be observed in patients whose neurologic exam is otherwise consistent with brain death. For example, a seesaw eye movement pattern was reported in a case with large cerebellar hemorrhage and fixed, dilated pupils (88), emphasizing that some residual brainstem neuronal activity may be shown by patients who otherwise meet the diagnostic criteria for brain death (8).

## 3 Quantitative ocular motor and vestibular testing in the unconscious patient

Quantitative ocular motor and vestibular testing offers the advantage of collecting standardized and reproducible eye movement responses and thus facilitating diagnosis, and the assessment of disease progression and their response to treatment (see also (11) for a review of the diagnostic and prognostic value of eye movement recordings in the unconscious patient). However, recording eye movements in the unresponsive patient in the ICU setting may be challenging or even not feasible (89). Both mobile equipment and better recording paradigms adapted to ICU setting are needed. Furthermore, with the ophthalmoscope one can detect pathological intrusive eye movements as small as a few tenths of a degree, emphasizing the importance of the clinical bedside examination.

For testing the integrity of the VOR both caloric irrigation (18) and (recently) galvanic stimulation have been used. Caloric irrigation applies a low-frequency stimulus to the vestibular organ and thus may elicit different responses compared to those from high-frequency head rotations. In deeply comatose patients combining head rotation with a caloric irrigation may be needed to elicit a vestibular response (12). Caloric irrigation also offers another way to stimulate the vestibular organs when head turns (as used for testing the VOR at the bedside) are contraindicated, e.g., in patients with spinal trauma.

The integrity of the VOR has been quantified in both comatose patients and patients in UWS (48). Both comatose patients and those in UWS showed an initial oppositely directed nearly compensatory eye movement when a fast head turn was applied. In both patient groups the head was then kept in a position such that the eyes were brought to an eccentric position. With this paradigm eccentric gaze holding was impaired in both groups; the eyes drifted back toward the center position indicating the velocity-to-position integrator was impaired, which was likely due to loss of cerebellar Purkinje cells. Sinusoidal oscillations of the head produced compensatory slow phases in both patient groups. Intermittent quick phases, however, were seen only in patients with UWS and were not sufficient to keep the eyes close to the straight-ahead position in the orbit.

### 3.1 Caloric irrigation

Cold-water caloric irrigation has been a prognostic tool in the unconscious patient for decades. Key parameters in studies focusing on the caloric-vestibular response were whether the eyes shifted towards the stimulated side during cold-water caloric irrigation and whether there was a fast-phase of the nystagmus. In the unconscious patient no quick phases of nystagmus are expected and absence of the tonic deviation may be of prognostic value. For example, caloric irrigation was used to predict brain death in 60 comatose patients, distinguishing a preserved tonic deviation from a present but abnormal response (with irregular, disconjugate eye movements) and an absent response in one study (90). While recovery rates were 42 and 9% in those with a preserved tonic deviation or a present but abnormal response, respectively, a 100% brain death rate was noted in the group with absent VOR during caloric irrigation (90). Likewise, from 100 unconscious patients that suffered TBI, an absent caloric-vestibular response 1–3 days after the head trauma was predictive for poor outcome with death in all patients (91). In contrast, those with a (partial) response demonstrated better outcome, but there was no relation between the VOR pattern and the duration of unconsciousness (91). In another study with 42 patients that suffered severe TBI, a correlation between the caloric-vestibular response and the state of consciousness was confirmed (92). Importantly, brain death should never be diagnosed solely on caloric responses, but always in conjunction with accepted criteria based on other assessments of brain function (8).

Caloric irrigation may help detect damage to the brainstem, with responses usually being absent (93). However, in patients with diffuse neocortical brain damage (e.g., due to hypoxic encephalopathy), brainstem reflexes including the VOR may be preserved despite an isoelectric EEG (94). Thus, depending on the underlying cause of coma, EEG, responses to head rotation, and responses to caloric irrigations may not correlate. However, when the caloric response is absent, it confirmed brain death as did the isoelectric EEG in the same patients (95). Importantly, compared to bedside VOR testing, caloric irrigation may be more sensitive in eliciting reflexive eye movements, as demonstrated for example in 81 comatose patients from various causes. Specifically, from 25 patients with either absent or inconclusive VOR (as tested by applying rapid passive head turns) at the bedside, a caloric response could be seen using ice-water (19). Overall, 92% of all patients with an abolished VOR at the bedside and an abolished caloric- response eventually died in this cohort, whereas 67% of those with preserved caloric responses had a good outcome. The discrepancy between bedside VOR response to head rotation and response to caloric irrigation could be due to the different stimulus frequencies (high vs. low frequency). Noteworthy, absent caloric responses do not always predict poor outcome, as emphasized by others. Specifically, 11 out of 46 deep comatose patients that demonstrated no VOR response on caloric irrigation obtained as early as possible after admission, survived in either UWS (*n* = 7) or recovered (*n* = 4) (96). These studies underline the value of reflexive eye movements in outcome prediction in the comatose patient, but also identify limitations and exceptions that need to be kept in mind.

In patients with UWS, the value of bedside caloric testing in predicting consciousness recovery was also studied (97). In 26 patients with UWS (mainly due to hypoxic encephalopathy [23/26 cases]), the sensitivity of the presence of the fast-component of nystagmus during VOR testing to predict recovery of consciousness was 1.00 and the specificity reached 0.92. Thus, the presence of both the slow tonic drift and the fast-component of the nystagmus was considered valuable in predicting outcome and also supported decisions about diagnostic work-up and treatment. In a follow-up study from the same group, the presence of a fast-phase of nystagmus during caloric irrigation in unconscious and mechanically ventilated patients without brainstem or cranial nerve lesions supported a favorable outcome (89).

### 3.2 Galvanic stimulation

Galvanic vestibular stimulation has been evaluated as a potential substitute for caloric irrigation to assess vestibular function in comatose patients, especially if caloric irrigation was contraindicated (e.g., because of rupture of the tympanic membrane or fractures of the petrous bone). By placing a pair of electrodes attached between the mastoid and the interscapular region, the right and left labyrinth could be stimulated independently (22). In a series of 10 comatose patients with absent bedside VOR responses, vestibular responses could be demonstrated using galvanic stimulation and caloric irrigation (98). In a proof-of-concept study assessing the value of predicting outcome, galvanic vestibular stimulation was preserved in four patients that survived with deficits ranging from UWS to moderate disability, whereas no response could be elicited in a fifth patient that was diagnosed brain-death 2 days later (22). In patients with TBI, ocular motor responses to early galvanic vestibular stimulation were helpful in predicting favorable outcome (99).

### 3.3 Visual fixation and visual pursuit assessments

Visual following of moving stimuli is mediated by cortical networks and its presence is considered sufficient for diagnosing MCS (4), whereas visual fixation (i.e., a movement of the eyes from an initial fixation point with a re-fixation on the new target location for more than 2 s) is considered a doubtful behavioral sign to discriminate UWS from MCS (100, 101). Visual stimuli to elicit visual tracking may include responses to motion of a finger, face, mirror or an optokinetic drum. Amongst those objects, detection rates of visual pursuit were highest for use of a large mirror (covering most of the patient’s visual field), which is also known for its ability to engage strong attention in healthy human subjects (102). Its presence is considered a key clinical marker of evolution from UWS (pursuit observed in 20% of patients) to a MCS (with pursuit seen in >80% of patients) (103). When reappearing after severe brain damage, this supports a favorable outcome, with recovery of consciousness in 73% compared to 45% when this finding was absent (104). However, at the bedside its detection varies depending on inter-rater reliability, within-day fluctuations and assessment tools (4). This can be overcome by use of quantitative eye movement tracking for detecting visual fixation and visual pursuit (105). Visual tracking (or visual pursuit) was studied for predicting outcome in unconscious patients due to various reasons. For example, video-eye tracking was applied to 10 unresponsive patients after TBI and compared to clinical eye-tracking assessment, demonstrating a higher rate of eye tracking detection using VOG and a higher probability of recovery in those that showed visual tracking (4/4) compared to those that did not (2/6) in one study (106). Thus, the authors concluded that preserved video-eye tracking may indicate a MCS.

## 4 The value of ocular motor and vestibular measures in assessing the prognosis

Developing predictive measures for recovery from coma and UWS is an unmet need and of great importance both from health, ethical and economic perspectives. Various potential predictors for poor outcome in comatose survivors after cardiac arrest have been systematically reviewed recently. However, no assessment of the potential role of eye movement analysis was provided and no use of machine learning / artificial intelligence was made in these studies (107). Thus, few studies have assessed the prognostic value of ocular motor and vestibular assessments in unconscious patients in a prospective way. This, however, is a prerequisite for the design of longitudinal studies in these patient populations. Feasibility studies have suggested that quantitative eye tracking in combination with machine-learning algorithms may serve as a non-invasive biomarker for recovery after TBI (106). Likewise, in a prospective study following up the natural history of patients with non-traumatic coma, the presence of orienting eye movements and of the VOR at the bedside were among the features predicting for reaching independent existence (108), while those patients with absent VOR or absent ocular following had poor outcomes. In comatose patients after cardiac arrest, assessing eye movements at the bedside could be used to help predict outcome (109). A lack of a VOR response or a lack of spontaneous orienting or conjugate roving eye movements within 2 weeks were poor prognostic markers.

The potential value of eye movements in predicting recovery was investigated in patients with UWS as well. In one study, EEG features (gamma power modulation) in association with what were described as “conjugate slow ballistic eye movements” have been quantified to develop a potential method to detect signs of cortical responsiveness (110). In this study, changes in the time course of gamma power in relation to conjugate eye movements were observed only in less severely affected UWS patients. However, the question of whether this pattern is supportive of recovery in UWS patients remains to be addressed.

## 5 Current limitations and future directions of eye movement measures in the unconscious patient

The lack of standardized, quantitative ocular motor and vestibular testing in the unconscious patient is a major deficiency in the research of unconscious patients. Likewise, no clear and consensus-driven semiological classification for various eye movement abnormalities in the unconscious patient is available, making a uniform description of eye movement patterns seem challenging.

Furthermore, the spectrum of underlying causes of unconsciousness is broad and strongly affects the prognosis. Incomplete descriptions of the extent of brain damage both structurally and functionally and the use of sedative and other drugs that affect brain function further add variability when assessing patient cohorts for outcome and features with predictive power. For bedside assessments of spontaneous and reflexive eye movements, only moderate inter-rater agreement was noted (with Kappa-values between 0.46 and 0.49) (111), emphasizing the need for appropriate training in assessing eye movements in the comatose patient and also for quantitative measurements. The availability of mobile VOG devices with virtual reality (VR) environments allows the application of a broad range of visual and vestibular stimuli in the ICU patient in a standardized way and thus presents a promising technological development. However, the difficulty of accurate calibration remains a challenge to obtaining reliable quantitative eye movement recordings in the unconscious patient. Finally artificial intelligence (AI) offers a great opportunity to analyze large, continuous data streams from sensors including electrocardiography, EEG and others as collected in ICU patients in a more meaningful and efficient way. Such an example is training a machine learning algorithm to detect eye movements from continuous EEG traces in comatose patients after cardiac arrest for optimizing prognostication. The feasibility of this approach was demonstrated, with high accuracy of automated eye movement detection from electro-oculography (EOG) in post cardiac-arrest patients in one study (112). EEG has the advantage of its broad availability in ICUs, standardized recording setups and data collection. However, the value of such novel approaches must be evaluated in the clinical setting. Big data analytics will facilitate the identification of clusters, thus, future multicenter studies with large sample sizes will allow a better description of patterns of eye movement abnormalities in unconscious patients Furthermore, our understand of disease mechanisms causing eye movement abnormalities has grown substantially in the past decades and new testing paradigms (such as positional testing and quantitative assessment of ocular torsion) have been added and allow for a more detailed interpretation of findings.

## 6 Conclusion

Eye movement testing in the unresponsive patient, especially caloric and “dolls head” VOR responses to head rotations, has been a bedrock of the neurological approach to the comatose patient for more than half a century. It offers targeted bedside testing of several brainstem reflexes and the evaluation of the integrity of neuronal networks connecting the cortex, brainstem and the cerebellum. Modern ways to record eye movements at the bedside with video-oculographic techniques and virtual reality, refinements of the evaluation and better understanding of vestibular responses, and new testing techniques such as galvanic stimulation, detection of torsional eye movements, and vestibular evoked potentials, all provide new ways to probe the function of the brain and correlate findings with cause, treatment and outcome. Analysis of patterns of eye movements plays a growing role in determining the prognosis of unconscious patients. Specifically, the presence of reflexive eye movements such as the VOR or visual following has been linked to better outcome and therefore should be included when assessing outcome. Quantitative, non-invasive continuous eye tracking, combined with AI-based analysis of large, multimodal datasets collected in a modern ICU may be used as a tool for prognosis in the future.
